# Repeated cycles of *Clostridium*-directed enzyme prodrug therapy result in sustained antitumour effects *in vivo*

**DOI:** 10.1038/sj.bjc.6603367

**Published:** 2006-10-03

**Authors:** J Theys, O Pennington, L Dubois, G Anlezark, T Vaughan, A Mengesha, W Landuyt, J Anné, P J Burke, P Dûrre, B G Wouters, N P Minton, P Lambin

**Affiliations:** 1 Department of Radiation Oncology (Maastro Lab), GROW Research Institute, University of Maastricht, UNS 50/23, PO Box 616, Maastricht 6200 MD, The Netherlands; 2 Institute of Infection, Immunity and Inflammation, Centre for Biomolecular Sciences, University of Nottingham, University Park, Nottingham NG7 2RD, UK; 3 Experimental Radiobiology/LEO, Gasthuisberg-CDG, Herestraat 49, Leuven 3000, Belgium; 4 Laboratory of Bacteriology, Rega Institute, KU Leuven, Minderbroedersstraat 10, Leuven 3000, Belgium; 5 Enact Pharma, Porton Down Science Park, Salisbury SP4 0JG, UK; 6 Centre for Applied Microbiology and Research, Porton Down, Salisbury SP4 0JG, UK; 7 Mikrobiologie und Biotechnologie, University Ulm, Ulm 89069, Germany

**Keywords:** *Clostridium*, hypoxia, conjugation, nitroreductase, tumour

## Abstract

The unique properties of the tumour microenvironment can be exploited by using recombinant anaerobic clostridial spores as highly selective gene delivery vectors. Although several recombinant *Clostridium* species have been generated during the past decade, their efficacy has been limited. Our goal was to substantially improve the prospects of clostridia as a gene delivery vector. Therefore, we have assessed a series of nitroreductase (NTR) enzymes for their capacity to convert the innocuous CB1954 prodrug to its toxic derivative. Among the enzymes tested, one showed superior prodrug turnover characteristics. In addition, we established an efficient gene transfer procedure, based on conjugation, which allows for the first time genetic engineering of *Clostridium* strains with superior tumour colonisation properties with high success rates. This conjugation procedure was subsequently used to create a recombinant *C. sporogenes* overexpressing the isolated NTR enzyme. Finally, analogous to a clinical setting situation, we have tested the effect of *multiple* consecutive treatment cycles, with antibiotic bacterial clearance between cycles. Importantly, this regimen demonstrated that intravenously administered spores of NTR-recombinant *C. sporogenes* produced significant antitumour efficacy when combined with prodrug administration.

Tumour heterogeneity, particularly with regard to hypoxia and necrosis, can dramatically limit the effectiveness of anticancer therapies. Recent analysis has shown that even in small distant metastasis, large avascular regions are present which can constitute between 25 and 75% of the tumour mass (Dang *et al*, 2001). The poorly vascularised, hypoxic cells adjacent to these areas are difficult to eradicate with conventional treatments and can also negatively influence the efficacy of novel treatment strategies. In that context, the limited success of initially exciting oncolytic adenoviral vectors may be attributed to hypoxia, as the hypoxia-induced G1 arrest of cells is responsible for lack of viral replication, thereby rendering these vectors less effective for anticancer therapy (Pipiya *et al*, 2005; Shen and Hermiston, 2005). Similarly, retroviral vectors might also be less effective in hypoxic conditions, since upon activation of the PERK kinase under hypoxia, eIF2*α* gets phosphorylated, leading to overall inhibition of translation (Koumenis *et al*, 2002). This defence mechanism against viral infection and the fact that hypoxic cells do not proliferate can negatively influence retroviral efficiency under hypoxic conditions. Perversely, the very existence of these hypoxic/necrotic regions may provide the solution. This is because they provide rather ideal environments for the growth and proliferation of obligate, anaerobic bacteria. Thus, while intravenously injected clostridial spores are dispersed throughout the body, only those that encounter the hypoxic environment of a solid tumour go on to germinate and multiply (Mose and Mose, 1964; Carey *et al*, 1967). Delivery is exquisitely selective, and has led to the suggestion that clostridia could be used as tumour-specific vectors for therapeutic gene delivery (Minton *et al*, 1995; Fox *et al*, 1996; Lemmon *et al*, 1997; Lambin *et al*, 1998).

During the past decade, several recombinant clostridial species that can express therapeutic proteins specifically in tumours have been generated (Lemmon *et al*, 1997; Theys *et al*, 2001a; Liu *et al*, 2002; Barbe *et al*, 2005). Although these reports established the feasibility of this delivery system and showed some antitumoural promise, the properties of the strains used were suboptimal, particularly with respect to tumour colonisation. So far, clostridial strains able to more effectively colonise tumours, typified by *Clostridium sporogenes*, have proven largely recalcitrant to DNA transfer.

Until now, the principal therapeutic proteins delivered using clostridial spores have been prodrug converting enzymes (Minton, 2003). These enzymes are characterised by their bystander effect and form the basis of the *Clostridium* directed-enzyme-prodrug therapy (CDEPT). In this context, the nitroreductase (NTR) class of enzymes are of particular interest because their small size increases the likelihood of efficient clostridial expression. Nitroreductase converts the 4-nitrogroup of the prodrug CB1954 (5-aziridinyl-2,4-dinitrobenzamide) to its 10 000-fold more toxic 4-hydroxylamine (4HX) derivative, which can be further metabolised to form a DNA–DNA crosslinking and apoptosis-inducing agent (Palmer *et al*, 2003). Interestingly, both proliferating and nonproliferating cells, as they are often present in tumour areas with gradients of hypoxia, are killed (Cui *et al*, 1999). The initially detected rat enzyme (DT-diaphorase), has been superseded by an enzyme (NTR-B) isolated from *Escherichia coli* B, due to its increased activity against CB 1954. However, unlike the rat enzyme, NTR-B also reduces the 2-nitrogroup to produce the relatively nontoxic 2-hydroxylamine (2HX) derivative.

Our goal was to substantially improve the prospects of clostridia by several ways. Firstly, we aimed to improve the enzymatic conversion of the CB1954 prodrug by isolating a new NTR enzyme with equivalent or better kinetic parameters to NTR-B, but which produced only the 4HX derivative of CB 1954. Secondly, we sought to increase the levels of bacteria, and inherently of therapeutic protein, in the tumour. This required the development of a method that allowed efficient gene transfer to strains with enhanced tumour colonising capacities such as *C. sporogenes*. Finally, we intended to evaluate in a quantitative way the ability of the therapeutic protein delivered by the recombinant clostridia to convert sufficient levels of prodrug to produce *in vivo* antitumour efficacy. To this end, we limited the period of active prodrug conversion by the metabolically active vegetative recombinant clostridia in the tumour to a 2-week period by administration of antibiotics. Analogous to a clinical setting situation, we used this approach to test the effect of *multiple* consecutive treatment cycles.

## MATERIALS AND METHODS

### Bacteria and plasmids

*C. sporogenes* NCIMB 10696 was obtained from the National Collection of Industrial and Marine Bacteria (NCIMB Ltd, Aberdeen, Scotland). The strain was grown in TYG media (30 g l^−1^ tryptone, 20 g l^−1^ yeast extract powder, 1 g l^−1^ sodium thioglycollate supplemented with 250 *μ*g ml^−1^ D-cycloserine (to select for *C. sporogenes*)) at 37°C in an MkIII anaerobic workstation (Don Whitley, Shipley, UK) with 80% N_2_, 10% H_2_, 10% CO_2_ atmosphere. For general cloning, *E. coli* TOP10 (Invitrogen, Paisley, UK) was used. The host employed for expression studies was NovaBlue (DE3). Strains were grown in Luria-Bertani media at 37°C. Media were supplemented, when applicable, with erythromycin (500 *μ*g ml^−1^ in *E. coli* or 10 *μ*g ml^−1^ in *C. sporogenes*), ampicillin (100 *μ*g ml^−1^), tetracycline (15 *μ*g ml^−1^) or 5-bromo-4-chloro-3-indolyl-*β*-galactoside (X-Gal; 40 *μ*g ml^−1^). Plasmids used in this study are listed in Table 1.

### Isolation of NTR enzymes

Homologues to the previously isolated (Michael *et al*, 1994; Anlezark *et al*, 2002) bacterial NTRs of *E. coli* (NfnB) and *Bacillus amyloliquefaciens* (YwrO) were identified in bacterial genomes using BLASTP. A total of 10 NfnB-like proteins and five YwrO-like proteins were selected for further study. DNA encompassing each encoding gene was amplified from the relevant genomic DNA of the target organism by PCR using appropriate oligonucleotide primers and cloned into the expression vector pET21b. In each case, the 5′-primer was designed so that it incorporated an *Nde*I restriction site (CATATG), whereby the ATG corresponded to the translational start codon of the gene. Each gene was inserted into pET21b at the *Nde*I site such that translational start codon was placed at the optimal distance from the vector encoded ribosome-binding site (RBS). To purify the enzyme, *E. coli* NovaBlue (DE3) cells carrying the wild-type (wt) gene were grown overnight at 37°C following induction with IPTG. Recombinant NTR enzymes were purified to homogeneity as previously described (Anlezark *et al*, 2002).

### NTR assays

Quantitative assays using CB 1954 substrate were carried out at 37°C by HPLC as previously described (Anlezark *et al*, 2002). When qualitative assays were used to identify column fractions, the standard conditions were 1 mM prodrug, 2 mM NAD(P)H, 4% DMSO in 100 mM sodium phosphate buffer pH 7, 37°C. Incubation times varied according to the enzyme activity being studied. Assays using menadione as substrate were carried out spectrophotometrically as previously described (Knox *et al*, 1988) using cytochrome *c* as terminal electron acceptor. Similar procedures were used to assay flavin reductase activity with FMN and FAD as substrate and with cofactors NADH and/or NADPH. Kinetic parameters with respect to CB 1954 were determined by HPLC assay using 10 *μ*l of the final product in 500 *μ*l assay mix containing 100–1000 *μ*M CB 1954, 500 *μ*M NADPH and incubating the mixture at 37°C for 10 min. Reduction of CB 1954 was determined by comparison of peak areas at 325 nm in standard and enzyme tubes. Enzyme kinetics were only undertaken on those enzymes found in qualitative assay to produce predominantly the 4-hydroxlamine derivative.

### *In vitro* cytotoxicity of CB1954

Microtitre plates (96 well) were obtained preseeded with V79 cells at 10 000 cells ml^−1^ (European Collection of Animal Cell Cultures, ECACC) in DMEM+10% FCS. CB 1954 was dissolved in DMSO (Sigma, Gillingham, Dorset SP84XT, UK, tissue culture grade) so that the appropriate concentrations could be dispensed by adding 5 *μ*l per well. NAD(P)H was dissolved in sterile PBS to give the appropriate final concentration by adding 10 *μ*l per well. Enzymes were diluted in sterile PBS. The cells were exposed for 3 h to CB 1954 or SN 23862 (3.9–500 *μ*M in doubling dilutions) alone or in combination with cofactor (NAD(P)H 125 or 250 *μ*M) and enzyme (4 *μ*g) and subsequently left to incubate at 37°C and 5% CO_2_ for 3–4 days. Cytotoxicity was quantified by sulphorhodamine B (SRB) assay. Briefly, cells were fixed by adding cold 10% TCA for 30 min and washed before adding 0.4% dye in 1% acetic acid and incubating at room temperature for 30 min. After washing and air drying at room temperature the dye was solubilised by adding 100 *μ*l of 10 mM Tris to each well. The plates were read at 492 nm in a Titertek plate reader. Cytotoxicity towards treated cells was expressed as % of A_492_ of untreated controls and statistical analysis was performed using the Mann–Whitney test. ED_50_s were calculated using probit analysis.

### Construction of the clostridial expression vectors

The erythromycin resistance gene (*ermB*) from pMTL20E was PCR amplified using the primers ermBF (5′-ATGACTGATATCACT GATGCTAGCGAAATGATACACCAATCAG-3′) and ermBR (5′-CTTAGTGTTAACACAGCTGTAGGCGCTAGGGACCTC-3′), and cloned into pMTL4 digested with *Eco*RV to generate pMTL4E. The Gram-positive replicon from pIMP1 was cloned between the blunt-ended *Bsp*HI sites of pMTL4E, deleting the *bla* gene in the process. The plasmid obtained was designated pMTL552. The expression cartridge was obtained from pMTL9341aLS2 and modified encompassing an altered ferredoxin promoter (fac2) in which the sequence preceding the ATG start codon had been replaced with AGGAGGTTAGTCAT, such that the RBS (AGGAGG) was 8 bp away from the ATG start. The entire modified expression cartridge was then cloned into the *Eco*RV site of pMTL552, to give pMTL553. For conjugative mobilisation of the plasmid, a *Sma*I/*Eco*RV fragment carrying the RK2 OriT region was isolated from the plasmid pEOriT and inserted into the *Pvu*II site of pMTL553 yielding pMTL554. To enable blue/white selection for cloning purposes, LacZ alpha was cloned in pMTL554 to yield the final expression vector, pMTL555.

The most effective NTR enzyme identified was YC78_HAEIN of *Haemophilus influenzae*, accession Q57431, annotated as a putative NAD(P)H NTR. A synthetic gene encoding this protein was synthesised (Entelechon Gmbh, Germany), incorporating typical *Clostridium* codons. In parallel, the wt gene was amplified from the *H. influenzae* chromosome using the primers HinNTRF (5′-GAGGAAATCATATGACTCAAC-3′) and HinNTRR (5′-CTGCAGGCCTTTTTTAAT-3′). Both the wt and synthetic gene were inserted into pMTL555 between the *Nde*I and *Pst*I sites to yield pOJP10 and pOJP11, respectively.

### Conjugation procedure

Plasmids were introduced into *C. sporogenes* NCIMB 10696, M-55 and *Clostridium novyi-NT* (Dang *et al*, 2001) using a modification of the protocol previously described by Purdy *et al* (2002). Briefly, cells harvested from a 1 ml overnight culture of the *E. coli* donor were washed in PBS before being resuspended in 200 *μ*l of an overnight culture of *C. sporogenes* or *C. novyi-NT* grown in TYG broth. The 200 *μ*l mating mix was spotted onto a TYG+0.5% glucose (v v^−1^) agar plate and incubated anaerobically for 7 h. The mating mixture was subsequently resuspended in 500 *μ*l of sterile PBS before plating onto selective agar (TYG+erythromycin). *E. coli* donors were counterselected by the addition of D-cycloserine (250 *μ*g ml^−1^) to the media. Recombinant colonies of *C. sporogenes* NCIMB 10696 were screened for the presence of the plasmid by PCR and also by retransforming the plasmid back into *E. coli* followed by restriction digestion verification of the plasmid.

### *In vivo* evaluation of antitumour effect

Human colorectal carcinoma (HCT116) were injected subcutaneously (1.5 × 10^6^ cells) in the abdominal flank of female adult NMRI nu/nu mice. Tumours were measured at least twice/week in three orthogonal diameters and volumes calculated according the formula *A* × *B* × *C* × *π*/6. All experiments were conducted in accordance with local institutional guidelines, approved by the Animal Ethics Committee of the University and procedures were according to the guidelines defined by the UKCCCR (Workman *et al*, 1998).

When tumours reached an average volume of 400 mm^3^, treatment was started. Tumour colonisation was allowed f or 5 days before initiation of prodrug and sham treatment, while selection antibiotics (erythromycin at 60 mg l^−1^) were added to the drinking water. CB1954 prodrug (15 mg kg^−1^) was prepared as previously described (Djeha *et al*, 2000) and administrated intraperitoneally (i.p.) for 5 consecutive days. Animals were subsequently treated with 200 mg kg^−1^ Flagyl® i.p. twice daily for 9 days. During this period, the drinking water was also supplemented with Flagyl®. Body weight measurements were used as a parameter for treatment toxicity. At the end of the follow-up period, or when tumours outranged the ethically allowed maximal volume, animals were killed by cervical dislocation. Tumours and normal tissues (liver, spleen) were excised, grinded and examined for colonisation levels at different time points using a procedure described previously (Lambin *et al*, 1998).

### Statistics

All statistical analyses were performed with SPSS 12.0.1 for Windows (SPSS Inc., 2003, Chicago, IL, USA). Mixed models linear regression was used to determine the statistical significance of differences between two independent groups of variables.

## RESULTS

### Isolation of novel NTR enzyme

Recombinant procedures were used to produce purified protein from 10 bacterial homologues of the *E. coli* NfnB (NTR-B) enzyme (Michael *et al*, 1994) and five homologues of YwrO of *B. amyloliquefaciens* (Anlezark *et al*, 2002). Qualitative assays showed that only one of the YwrO-like enzymes and four of the NfnB homologues produced predominantly the 4HX derivative of CB1954. These enzymes were, therefore, subjected to quantitative analysis and their *K*_m_ and *k*_cat_ with CB1954 determined (Table 2). The enzyme NTR-H isolated from *H. influenzae* (Accession no. YP_249310) possessed the most favourable properties. In addition to producing only the 4HX derivative from CB1954, it had a *K*_m_ and *k*_cat_ for this substrate of 690 *μ*M and 56.2 s^−1^, respectively. This compares to the previously isolated bacterial enzymes of *E. coli* B (NTR-B) and *B. amyloliquefaciens* (YwrO) which possess a respective *K*_m_ of 862 and 618 *μ*M and a *k*_cat_ of 6.0 and 8.2 s^−1^. Thus, while the affinity of NTR-H and NTR-B for the prodrug substrate (*K*_m_) is broadly equivalent, the rate at which it is turned over (*k*_cat_) is an order of magnitude greater in the case of NTR-H. Moreover, the entire reaction product is the toxic 4HX derivative, as opposed to NTR-B where 50% of the reaction results in the nontoxic 2HX derivative.

**Figure 1:**
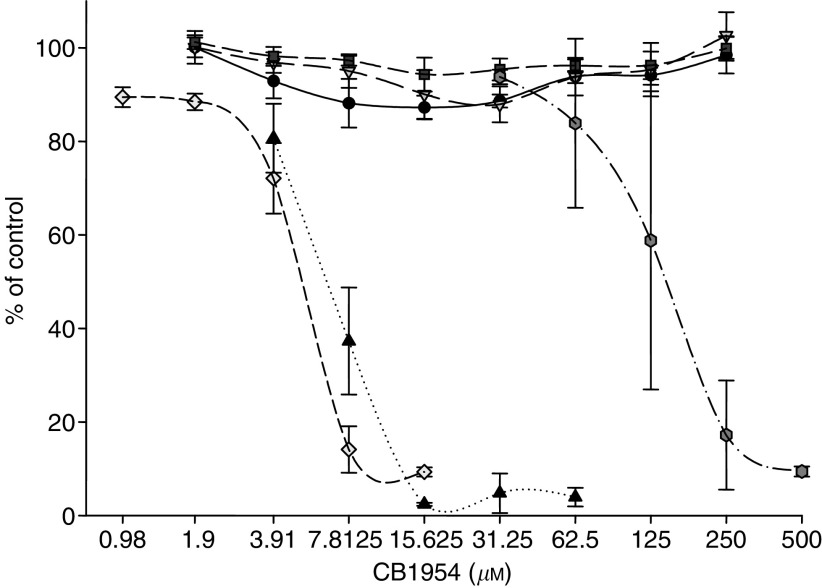
Assessment of CB1954 cytotoxicity. Cytotoxicity of CB 1954 was assessed in 96-well plates seeded with V79 cells (10^4^ per ml) by incubating them with CB 1954 alone (3.9–500 *μ*M), prodrug+NAD(P)H (250 *μ*M), or prodrug, cofactor and enzyme (4 *μ*g well^−1^) for 3 h in serum free DMEM. The medium was replaced and the cells grown for 72–96 h post-treatment in DMEM+10% FCS until control (untreated) cells had achieved confluence. The cells were fixed, stained with SRB and the plates read at 492 nm. Results are expressed as % of control (untreated cells). Key: •, prodrug alone; ○, prodrug +NADH; ▴, prodrug+NADPH; ▪, HinNTR+NAPDH+Prodrug; □, NfnB+NADH+prodrug, and; ♦, YwrO+NADPH+prodrug.

The benefits of these improved properties on cytotoxicity were demonstrated in a set of experiments in which equivalent quantities of purified NTR enzyme were incubated with V79 cells in the presence of varying concentrations of CB1954 (Figure 1). The greatest degree of killing was observed in the case of NTR-H. In general, with all the enzymes tested (Table 2), ED_50_ appeared to be more highly related to *k*_cat_ as opposed to *K*_m_, with NTR-H proving to be the most potent.

### Transformation of *Clostridum spp* with superior tumour colonising properties

Having established the superiority of the NTR-H, we sought to introduce an expression plasmid carrying the gene into the *Clostridium* strains with the best tumour colonising properties. A method of transforming the selected species, *C. sporogenes*, has previously been described (Liu *et al*, 2002). However, repeated attempts to obtain transformants with this method of two different *C. sporogenes* strains and of *C. novyi-NT* in our laboratories over a period of 2 years were unsuccessful. We therefore explored the use of a conjugative procedure, initially developed for the introduction of plasmids into *Clostridium difficile* (Purdy *et al*, 2002).

In our initial experiments we utilised *C. sporogenes* ATCC 13732 as the recipient, and we were able to show that plasmids could be consistently introduced at appreciable frequencies. The rate of transfer varied between 1.5 × 10^−7^ and approaching 1.0 × 10^−4^ transconjugants per recipient, dependant on the plasmid employed (Table 3). Significantly, for those plasmids tested (Table 3) this method also proved to be applicable to *C. sporogenes* M-55 (Mose and Mose, 1964) and *C. novyi-NT* (Dang *et al*, 2001; Bettegowda *et al*, 2003), which had previously proven recalcitrant to all transformation attempts. With this conjugation method, we obtained success rates of 70–90%, depending on the strain being used. In all cases, the plasmid could be reisolated by transformation into *E. coli*, and its authenticity established through analysis of suitably restricted samples on agarose gels.

**Figure 2:**
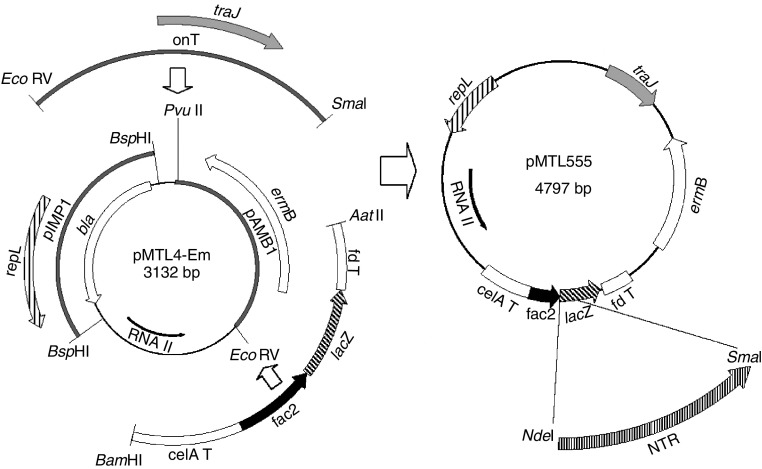
Construction of plasmids overexpressing NTR. The derivation of DNA fragments used to assemble the expression vector pMTL555 is as indicated. For a detailed description see Materials and Methods. Key: *repL*, replication protein of pIM13, taken from the derivative plasmid pIMP1, *bla*, *β*-lactamase of pMTL4; *ermB*, erythromycin resistance gene from plasmid pAM*β*1; *traJ,* transfer protein from the ‘origin of transfer’ (*oriT*) of plasmid RK2; RNAIII, replication region of ColE1, taken from the derivative plasmid pMTL4; celA T, transcriptional terminator of the *Clostridium thermocellum celA* gene; fac2, the promoter of the *Clostridium pasteurianum* ferredoxin gene, derivatised to include an *E. coli lac* operator; lacZ, *β*-galactosidase alpha fragment; fd T, transcriptional terminator of the *C. pasteurianum* ferredoxin gene. Gene fragments encompassing NTR enzymes (NRase) are cloned into the lacZ region using the *Nde*I site adjacent to the fac2 RBS and an appropriate site (e.g., *Sma*I) in the pMTL20 polylinker region present in lacZ.

Having derived an efficient gene transfer procedure, we constructed a purpose built expression vector, pMTL555, in which two different genes encoding NTR-H were independently cloned (Figure 2). The first represented the wt gene, the second was a synthetic gene, in which the codons were changed to match the *Clostridium* codon usage. Following the introduction of the two recombinant plasmids (pOJP10 and pOJP11, wt and synthetic NTR-H, respectively), verification of the transconjugants indicated no structural change to either pOJP10 or pOJP11. The levels of NTR in both recombinant derived samples were significantly higher compared to wt control samples. As the lysate derived from cells carrying pOJP11 produced slightly higher levels than cells harbouring pOJP10, we chose the NTR-H expressing *C. sporogenes* strain carrying pOJP11 to evaluate its ability to produce antitumour activity.

### *In vivo* antitumour activity following systemic administration of recombinant *C. sporogenes*

Our aim was to determine whether the levels of NTR-H delivered by the recombinant *C. sporogenes* were sufficiently high to cause enough prodrug conversion to result in measurable antitumour efficacy. A tumour regrowth delay assay was used to quantify the magnitude of this effect. Nu/nu mice xenografted with HCT116 were injected with NTR-H expressing *C. sporogenes* spores when tumours reached an average volume of 400 mm^3^. In order to be potentially useful for the treatment of disseminated or inaccessible tumours, injections were given systemically rather than through local injection. Animals were divided into four groups that received (1) no treatment, (2) CB1954 prodrug alone (15 mg kg^−1^, five times a week), (3) recombinant spores (at a concentration of 5.10^7^ cfu in a volume of 100 *μ*l saline via the tail vein) followed by prodrug vehicle only (sham treatment) or (4) recombinant spores followed by CB1954 prodrug solution, respectively. To allow tumour colonisation to take place, prodrug or sham treatment started at day 5 after spore injection. Although the *in vivo* feasibility of the CDEPT approach has been investigated in the past, it has been difficult to discriminate between the specific effect of prodrug conversion and the effect of the bacteria only. The main reason is the continuous presence of recombinant bacteria during the entire follow-up and consequently, their potential contribution to the observed antitumour effects. Therefore, in order to quantitatively evaluate the CDEPT approach, we eradicated the *C. sporogenes* form the tumour following the daily prodrug injections by an antibiotic treatment course (Flagyl®, 200 mg kg^−1^, 2 × daily, for 9 days). This allowed us to specifically separate the effects of the recombinant bacteria alone from the effects of the bacteria in combination with the prodrug.

**Figure 3:**
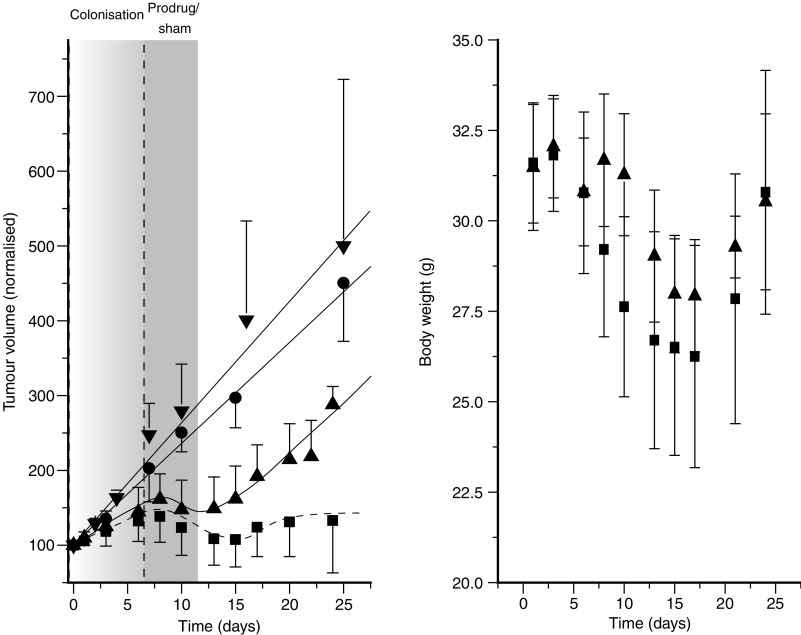
Antitumour effect following systemic administration of NTR-recombinant *C. sporogenes* spores to nu/nu mice bearing HCT116 tumours. (**A**) Tumour growth after one cycle of recombinant *C. sporogenes* treatment with CB1954 prodrug (▪) or vehicle only (sham) (▴) treatment. Control animals received no treatment (•) or CB1954 alone (▾). (**B**) Representative body weight evolution during a treatment cycle. Data are from at least five mice per group with error bars showing standard deviations for each group. Tumour volumes are normalised to 100 arbitrary units at the start of the treatment, allowing comparison of tumour growth within and across groups.

Tumour colonisation following recombinant spore administration but before the onset of antibiotic treatment was quantified by performing dilution series of randomly selected tumours from spore-treated animals. All tumours investigated showed colonisation levels of 10^8^–10^9^ cfu g^−1^ tumour tissue. As expected, viable clostridia could not be detected in normal tissues. Importantly, tumour colonisation levels decreased below the detection limit following antibiotic treatment, indicating the efficacy of the Flagyl® therapy. As already well established using other NTR-directed gene therapy approaches (Djeha *et al*, 2001), CB1954 administration alone had no effect on tumour volumes (Figure 3A). Administration of spores alone caused a moderate, but significant tumour growth delay (*P*<0.0001, mixed linear regression analysis) (Figure 3A). This was a consequence of modest tumour lysis and the appearance of haemorrhagic necrosis, an observation also made when using other clostridial species such as *C. sporogenes* M55 and *C. novyi-NT* (Dang *et al*, 2001). When combined with CB1954 administration, the antitumour effect significantly increased (*P*<0.0001, mixed linear regression analysis) (Figure 3A), thereby establishing the *in vivo* treatment efficacy of the NTR CDEPT approach. During the treatment, animals in both treatment arms showed a transient weight loss. This effect was not significantly different between the two groups. Most importantly, the observed weight loss was totally reversible and the animals recovered completely following the antibiotic treatment (Figure 3B).

### *In vivo* efficacy of repeated CDEPT treatment cycles

**Figure 4:**
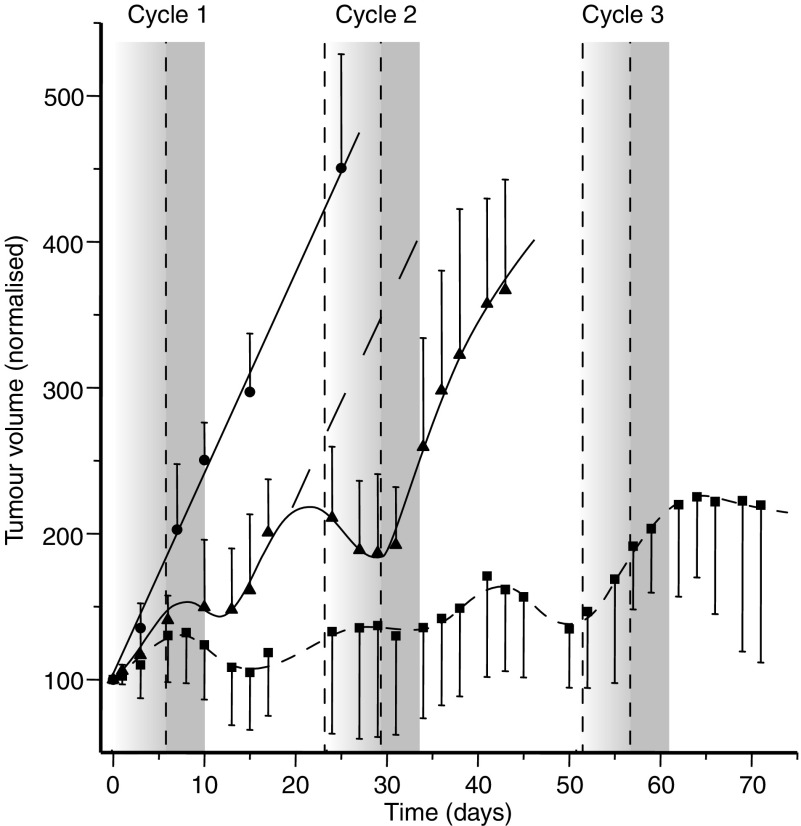
Antitumour effect following repeated treatment cycles of NRase-recombinant *C. sporogenes* in combination with CB1954 administration. Groups were recombinant *C. sporogenes* treatment with CB1954 prodrug (▪) or vehicle only (sham) (▴) treatment and control animals, receiving no treatment (•). The dashed line indicates the expected tumour growth following one treatment cycle (based on results shown in Figure 3A). A single treatment cycle consisted of bacterial spore administration at day 0, followed by CB1954/vehicle administration at day 6, during 5 days. Each treatment cycle was followed by antibiotic therapy and an additional recovery period for the animals (for more details, see legend Figure 3 and text). Sham-treated animals received two treatment cycles before tumour volumes outranged the ethically allowed maximum. The CB1954 treated group was given an additional third treatment cycle. Tumour volumes are normalised to 100 arbitrary units at the start of the treatment, allowing comparison of tumour growth within and across groups.

In a clinical setting, chemotherapy is typically given in multiple cycles in order to maximise its effects. Analogous to this situation, we questioned whether clostridial therapy could be given repeatedly. A treatment cycle started at the day of spore injection (day 1), followed 5 days later (day 6) by prodrug or vehicle treatment for 5 days. Each treatment cycle was then followed by antibiotic treatment during 9 days. Eventually animals were allowed to recover for an additional period of 5–7 days. Sham-treated animals were given two consecutive identical cycles. The tumour growth delay caused by each of the two (sham) treatment cycles was very similar (Figure 4). This result indicates not only that the bacteria can effectively recolonise the tumour to result in an antitumour effect, but also that the tumour microenvironment following the first treatment cycle did not fundamentally change. Despite the observed growth delay within the sham-treated group, the animals in this group had to be killed for ethical reasons by the end of the second treatment cycle because their tumour volumes became too high. In contrast, and more importantly, a highly significant and much bigger antitumour effect was observed in animals that were treated twice with recombinant spores combined with CB1954 prodrug (Figure 4). This allowed application of an additional third treatment cycle. Throughout the three cycles, the treatment resulted in sustained growth delay effects.

## DISCUSSION

In the current paper, we report significant progress in the use of clostridia to deliver prodrug-converting enzymes specifically to tumours. As a first step to improve the system, we isolated a novel NTR enzyme with better characteristics than NTR enzymes used so far. The NTR-H enzyme was demonstrated to be the best out of 15 tested bacterial NTR homologues. The enzyme produced only the toxic 4HX CB1954 derivative and showed the best kinetic parameters of all the enzymes tested. As expected, the superior enzyme properties translated into an enhanced toxicity profile *in vitro*. To evaluate the *in vivo* efficacy of this novel enzyme, transfer of the NTR-H enzyme to a *Clostridium* strain with enhanced tumour colonisation properties was absolutely necessary. Indeed, although previous CDEPT work undoubtedly established the safety and feasibility of the approach, proof of *in vivo* antitumour efficacy has so far been limited (Lemmon *et al*, 1997; Theys *et al*, 2001a; Liu *et al*, 2002). The major reason for this failure has been attributed to the low tumour colonisation efficiency of the employed strains. Until recently, the best tumour colonising strains, such as *C. sporogenes or C. novyi-NT*, could not be transformed. Thus, the reported development a few years ago of a transformation system based on electroporation for *C. sporogenes*, was encouraging (Liu *et al*, 2002). Unfortunately, the reported transformation procedure could never be reproduced in our laboratories. As exogenous endonucleases represent a major impediment to DNA transfer, we therefore devised a procedure based on conjugative transfer from *E. coli* donors. Here, we demonstrate that this procedure can be used to introduce recombinant plasmids into superior tumour colonising strains such as *C. sporogenes, C. oncolyticum* and *C. novyi-NT* at high frequencies and with high success rates. Thanks to this breakthrough, it will now be possible to express essentially any heterologous gene of interest in these clostridial vectors. As shown here for NTR-H, the adaptation of the heterologous gene codons to the codon usage in the clostridial host, characterised by its low G+C% content, might further increase therapeutic protein yield.

Finally, we evaluated if the recombinant *C. sporogenes* strain could deliver enough NTR-H to tumours *in vivo* to convert sufficient levels of prodrug to produce antitumour efficacy. Although *in vivo* antitumour effects using a CDEPT strategy have already been demonstrated with recombinant clostridia, it has been difficult to attribute the observed effects specifically to prodrug conversion, since the bacteria remain present in the tumour. As the bacteria have antitumour effects in their own right, as demonstrated in our work, it is not clear how much of the effect is due to their continuous presence. We therefore devised a way to quantify the magnitude of the antitumour effect due to prodrug conversion by giving pulse treatment cycles, followed by antibiotic treatment. Clearance of the bacteria from the animal not only allowed recovery after the treatment cycle, it also enabled a precise and direct quantification of the antitumour effect. In order to minimise all secondary effects that could be potentially involved in mediating the antitumour effect, we chose immunocompromised mice as a model, although there is no evidence for an immune response as shown earlier in immunocompetent rats (Theys *et al*, 2001b). Our results clearly demonstrate that intravenously administered spores of *C. sporogenes* expressing NTR-H produce significant antitumour efficacy when combined with prodrug administration. Most importantly, highly significant and prolonged antitumour efficacy could be obtained following repeated cycles. As we observe such a striking antitumour response, it must mean that not only the hypoxic but also aerobic cells are being killed as a result of an additional bystander effect. Such a phenomenon has indeed been described for NTR in combination with CB1954 (Djeha *et al*, 2000; Wilson *et al*, 2002). Therefore, the use of a prodrug activating enzyme not only is a further safeguard to the system (compared to expression of a direct cytotoxic protein which might produce host toxicity if it comes in circulation), but also provides considerable potential for treatment amplification. To our knowledge, this is the first demonstration of a sustained antitumour effect following multiple cycles of recombinant clostridia treatment. Overall, this indicates that maturation of inactive spores into vegetative rods following a first cycle does not eliminate the activity of subsequent administrations. Our data are in line with our own previously reported results (Theys *et al*, 2001b) and with the observations of Liu *et al* (2002) showing no reduction in numbers of vegetative cells g^−1^ tumour occurring between 7 and 14 days after a single spore injection. This is not limited to animals with an immunocompromised immune system since it was demonstrated some 25 years ago that repeated clostridial spore administration could be performed in tumour-bearing rats (Gericke *et al*, 1979). This is likely due to the lack of immunogenicity of the applied clostridial spores. Moreover, transition from spores to the vegetative reproductive state only occurs in the severe hypoxic and necrotic region of the tumour, considered to be an immune privileged site.

Although the observed effects of the therapy were significant, additional efficacy would be predicted when combined with radiotherapy and/or conventional chemotherapy. Other potential combined treatment modalities include specific targeting of the structurally abnormal tumour blood vessels with vascular targeting agents as we (Theys *et al*, 2001b) and others (Dang *et al*, 2001) have already successfully reported. In addition, more soluble derivatives of CB1954 that cause more effective tumour regression have recently been described (Wilson *et al*, 2002) and since 5-FU and CB1954 have been shown to act synergistically (Palmer *et al*, 2003), the combinatorial use of prodrug activating enzymes (CDase and NTR) might be a promising option for CDEPT.

## Figures and Tables

**Table 1:** Plasmids used in this study

**Plasmid**	**Features**	**Reference**
pMTL20E	Source of *ermB*	(Oultram *et al*, 1988)
pMTL4	Shuttle vector backbone	(Chambers *et al*, 1988)
pET21b	Expression vector	Novagen
pIMP1	pIM13 replicon	(Mermelstein *et al*, 1992)
pMTL6341aLS2	Clostridial expression cartridge	(Carter *et al*, 2005)
pMTL20	Cloning vector and source of LacZ*α*	
pMTL30	Source of OriT	(Williams *et al*, 1990)
pCR2.1-TOPO	TA PCR cloning vector	Invitrogen
pCR-Blunt II-TOPO	Blunt PCR cloning vector	Invitrogen
pCR4-TOPO∷fac2	Source of fac2 promoter	Entelechon GmBH
pMTL4-Em	Vector backbone – ColEI Gram-negative replicon+*ermB*	This study
pMTL552	pMTL4E+pIM13 replicon	This study
pMTL553	pMTL552+expression cartridge	This study
pMTL554	pMTL553+OriT	This study
pEOriT	OriT from pMTL30	This study
pMTL555	pMTL554+LacZalpha	This study

**Table 2:** Characteristics of purified bacterial nitroreductases

**Enzyme**	**Product**	***K*_m_ (*μ*M)**	***k*_cat_ (s^−1^)**	**ED_50_ CB 1954 (*μ*M)**
NfnB	4HX+2HX	682	6.0	6.3
YwrO BAM	4HX	617	8.2	137.1
YdgI	4>2HX	3863.9	30.3	15.3
YodC	4>2HX	552.2	58	20.3
*Haemophilus influenzae* NfnB	4HX	690	56.2	4.7
*Campylobacter jejuni* NfnB	4HX	217	6.1	55.8
*Porphyromonas gingivalis* YwrO	4HX	1200	3.2	252.3

4HX=4-hydroxylamine.

**Table 3:** Transfer frequencies of various plasmids from *E. coli* donors into three different clostridial strains

	**Conjugation efficiency (transconjugants per recipient strain)**		
**Plasmid (replicon)**	***C. sporogenes* ATCC 13732**	***C. sporogenes* M-55**	***C. novyi-NT***
pMTL555 (pIM13)	1.44 × 10^−7^	1.84 × 10^−7^	ND
pMTL9501/pCTC41 (pAM*β*1)	1.18 × 10^−6^	1.27 × 10^−6^	1.80 × 10^−7^
pMTL9401 (pCB102)	8.86 × 10^−5^	ND	ND
pMTL9611 (pIP404)	8.3 × 10^−6^	ND	ND
pMTL9301 (pCD6)	4.00 × 10^−5^	ND	ND

Plasmids pMTL9301, pMTL9401, pMTL9501 and pMTL9611 are described by Purdy *et al* (2002). Plasmid pCTC41 is described by Williams *et al* (1990).
